# Lipid nanoparticle encapsulated large peritoneal macrophages migrate to the lungs via the systemic circulation in a model of clodronate-mediated lung-resident macrophage depletion

**DOI:** 10.7150/thno.91062

**Published:** 2024-04-08

**Authors:** Dhaval Oza, Fernando Ivich, Joshua Pace, Mikyung Yu, Mark Niedre, Mansoor Amiji

**Affiliations:** 1 Department of Pharmaceutical Sciences, School of Pharmacy and Pharmaceutical Sciences, 360 Huntington Avenue, Northeastern University, Boston, MA 02115.; 2 Department of Bioengineering, College of Engineering, Northeastern University, 360 Huntington Avenue, Boston, MA 02115.; 3 Department of Chemical Engineering, College of Engineering, Northeastern University, 360 Huntington Avenue, Boston, MA 02115.; 4 Alnylam Pharmaceuticals, 675W Kendall St, Cambridge, MA, USA 02142.

**Keywords:** Peritoneal macrophages, GATA6 Transcription Factor, Small Interfering RNAs, Lipid Nanoparticles, C12-200, Diffuse *In Vivo* Flow Cytometry

## Abstract

**Rationale:** A mature tissue resident macrophage (TRM) population residing in the peritoneal cavity has been known for its unique ability to migrate to peritoneally located injured tissues and impart wound healing properties. Here, we sought to expand on this unique ability of large peritoneal macrophages (LPMs) by investigating whether these GATA6+ LPMs could also intravasate into systemic circulation and migrate to extra-peritoneally located lungs upon ablating lung-resident alveolar macrophages (AMs) by intranasally administered clodronate liposomes in mice.

**Methods:** C12-200 cationic lipidoid-based nanoparticles were employed to selectively deliver a small interfering RNA (siRNA)-targeting CD-45 labeled with a cyanine 5.5 (Cy5.5) dye to LPMs *in vivo* via intraperitoneal injection. We utilized a non-invasive optical technique called Diffuse* In Vivo* Flow Cytometry (DiFC) to then systemically track these LPMs in real time and paired it with more conventional techniques like flow cytometry and immunocytochemistry to initially confirm uptake of C12-200 encapsulated siRNA-Cy5.5 (siRNA-Cy5.5 (C12-200)) into LPMs, and further track them from the peritoneal cavity to the lungs in a mouse model of AM depletion incited by intranasally administered clodronate liposomes. Also, we stained for LPM-specific marker zinc-finger transcription factor GATA6 in harvested cells from biofluids like broncho-alveolar lavage as well as whole blood to probe for Cy5.5-labeled LPMs in the lungs as well as in systemic circulation.

**Results:** siRNA-Cy5.5 (C12-200) was robustly taken up by LPMs. Upon depletion of lung-resident AMs, these siRNA-Cy5.5 (C12-200) labeled LPMs rapidly migrated to the lungs via systemic circulation within 12-24 h. DiFC results showed that these LPMs intravasated from the peritoneal cavity and utilized a systemic route of migration. Moreover, immunocytochemical staining of zinc-finger transcription factor GATA6 further confirmed results from DiFC and flow cytometry, confirming the presence of siRNA-Cy5.5 (C12-200)-labeled LPMs in the peritoneum, whole blood and BALF only upon clodronate-administration.

**Conclusion:** Our results indicate for the very first time that selective tropism, migration, and infiltration of LPMs into extra-peritoneally located lungs was dependent on clodronate-mediated AM depletion. These results further open the possibility of therapeutically utilizing LPMs as delivery vehicles to carry nanoparticle-encapsulated oligonucleotide modalities to potentially address inflammatory diseases, infectious diseases and even cancer.

## Introduction

Macrophages are one of the most heterogenous, multi-functional and versatile cells of the innate immune system. They reside in almost every mammalian tissue and have well-established roles of maintaining tissue homeostasis and monitoring tissue microenvironment for infection and tissue damage 1-4. Tissue resident macrophages (TRMs) perform specialized functions and fulfil organ-specific roles reflected by their distinct transcriptomic profiles, plasticity, phenotype, and functionality across different tissues 1-8.

One such unique type of TRMs are the large resident macrophages of the peritoneal cavity 9. Novel findings about their behavior in context of acute tissue injuries have elucidated their tissue-specific functions and responses to injury stimuli 10. Based on their morphology, origin and phenotypic states, macrophages in the peritoneal cavity have been classified into monocyte-derived small peritoneal macrophages (SPMs) and tissue resident large peritoneal macrophages (LPMs), that are derived from embryogenic precursors 9, 10. What makes these 'cavity-associated' macrophages special is that although they have been categorized as tissue-resident, since they are not associated with any tissue per se, they can still contribute to the immunological responses of surrounding tissues, imparting wound-healing properties 11-16. Prior studies have shown that tissue-specific localization and functional polarization of LPMs can be driven by zinc-finger transcription factor GATA6, which is specifically expressed in LPMs among all TRMs 9, 10. Since then, multiple studies have confirmed that LPMs are the cell types within the peritoneal cavity that selectively expresses GATA6 11-16. Subsequently, it has been identified that GATA6 plays a vital role in LPM functionality and differentiation, and macrophage specific GAT6 KO studies in mice confirm increased rates of cell apoptosis pointing out to a vital role of GATA6 in LPM survival 17. Right from their adherence to the surrounding mesothelial lining in various pathological contexts, to migration to the omental 'milky spots', to even migrating outside the peritoneal cavity, a fairly context dependent migratory properties and functions of LPMs has been described 18-21.

Further exploration of this phenomenon more broadly across different tissues along with possible routes of migration to an extra-peritoneally located organ would expand these initial findings. Hence in this study, we focused on further diving into this unique feature of LPM migration to extra-peritoneally located lungs upon depleting out the lung-resident AMs and explore whether these cells intravasate and migrate via systemic circulation.

Alveolar macrophages (AMs) residing in the alveolar lumen of the lungs form the first line of defense for the respiratory tract 22-26. Here we demonstrate that selective depletion of AMs in the lungs via internasal administration of clodronate liposomes makes the LPMs more tropic towards the lungs and results in their infiltration 27-31. We specifically delivered a small interfering RNA (siRNA) labeled with fluorescent dye cyanine5.5 (Cy5.5) to LPMs using a promising lipid nanoparticle (LNP)-based delivery modality in C12-200-based LNPs (siRNA-Cy5.5 (C12-200)) 32-35. C12-200 is a novel 'lipidoid'-like ionizable lipid that has been characterized and used as the lipid of choice for immune-cell delivery of oligonucleotides in the past. C12-200-based LNPs have a primary mechanism of uptake by macrophage-induced phagocytosis 32. Owing to their prior success in achieving peritoneal macrophage selective uptake, we utilized these lipids to formulate siRNA encapsulating LNP 32. From our studies, we observe infiltration of labeled LPMs to the lungs 12-24 h following AM depletion, opening the question of how these macrophages residing in the peritoneum migrate to and infiltrate the lungs.

Furthermore, to explore whether LPMs migrate to the lungs via systemic circulation, we used an emerging technique - Diffuse *In Vivo* Flow Cytometry (DiFC) - to track migration of siRNA-Cy5.5 (C12-200) labeled LPMs to the lungs via the blood circulatory system 36, 37. DiFC allows non-invasive enumeration of fluorescent circulating cells without the need to draw blood samples 36, 37. It uses laser-induced fluorescence and highly scattered photons to detect moving cells and fluorescent sensors in relatively large, deeply seated blood vessels 36-39. As we show, DiFC revealed for the first time that LPMs migrate via the systemic circulation in the 12-24 h window following AM depletion, giving us deeper insights into route and kinetics of LPM migration post AM ablation.

In this study, we confirm a robust uptake of siRNA-Cy5.5 (C12-200) to LPMs and further identify that LPMs indeed show an inherent tropism towards the lungs upon ablation of AMs for the very first time. Moreover, we saw a fascinating dependency of intravasation of siRNA-Cy5.5 (C12-200)-labeled LPMs and lung infiltration on whether there was a depletion of lung-resident AMs or not. Together, these findings highlight a unique inherent property of a resident immune-cell type like LPM to migrate to non-peritoneally-located lung tissue and further broadens the potential of utilizing LPMs to deliver LNP-encapsulated siRNA payloads to injured tissue in future.

## Results

**Formulation of C12-200 lipid nanoparticle formulation and siRNA encapsulation:** Our main objective was to investigate whether LPMs could migrate to extra-peritoneally located organs like lungs by depletion of lung resident TRMs, and if so, whether they migrated via systemic circulation. To assess the selective tropism of LPMs in a clodronate-induced AM depletion model, we have utilized siRNA-encapsulated lipid nanoparticles. For all our studies, we utilized a CD-45-targeting siRNA Figure S1A) since CD-45 is a pan-macrophage surface marker. Hence, to track these cells, we labeled the sense strand of the siRNA with a Cy5.5 fluorophore (Figure S1A).

C12-200 LNP system is a four-compartment system with C12-200 along with helper lipids Distearoylphosphatidylcholine (DSPC), cholesterol and Poly(ethylene) glycol (PEG)-C14 and had a component molar ratio of ~50/10/38.5/1.5 (C12- 200/distearoylphosphatidylcholine/cholesterol/PEG-C14) (32-35 (Table 1). Synthesis of C12-200 and formulation of siRNA into the C12-200 was carried out as previously described 40. The final encapsulation efficiency (EE) of siRNA was 63% and the encapsulated siRNA concentration was 0.29 mg / ml. Average particle size was 70.93 nm, and the polydispersity index (PDI) of the final formulation was 0.048 (Table 1).

**Figure 1:**
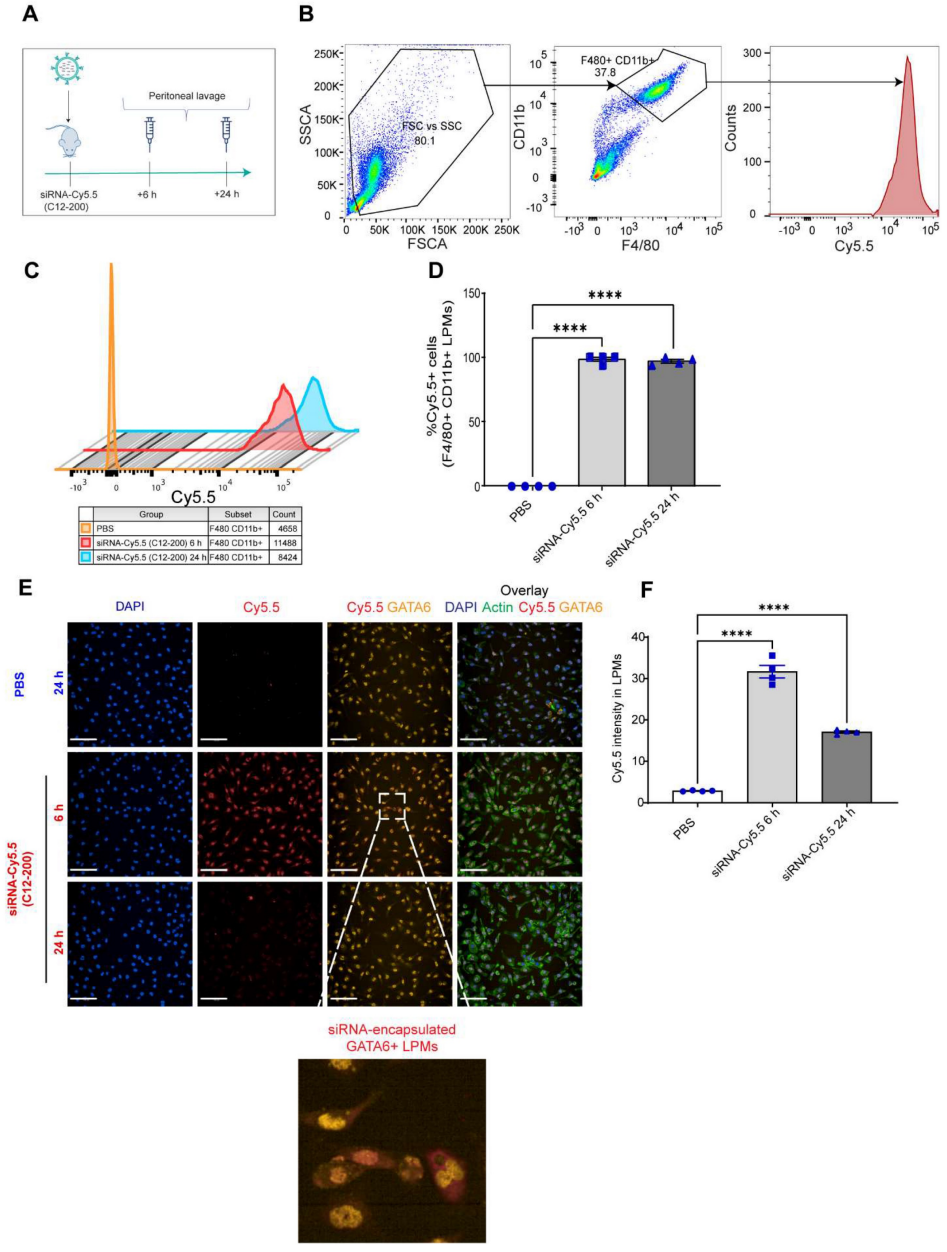
** siRNA-Cy5.5 (C12-200) are robustly taken up by LPMs. A.** Study schematics **B.** Representative flow cytometry gating and analysis of Cy5.5 MFI in peritoneal F4/80^hi^ CD11b^hi^ LPMs obtained from the respective treatment groups. Cells were pre-gated on size and viability. Data is representative of one sample from an n = 4 per treatment group. **C.** Representative histograms depicting the average Cy5.5 MFI from an n = 4 of respective treatment groups.** D.** Quantification of MFI of Cy5.5 in LPMs with the indicated groups. n=4 for all groups. **E.** Representative immunofluorescence images of siRNA-Cy5.5 (C12-200) (red) uptake in GATA6 positive (yellow) LPMs after isolating peritoneal lavage 6 h and 24 h post intraperitoneal administration. PBS-control mice were treated for 24 h. Scale bars, 200 µm. **F.** Quantification of Cy5.5 intensity of isolated GATA6+ LPMs in all the treatment groups. Data has been represented as Mean +/- SEM *p < 0.05, **p < 0.01, ***p < 0.001, **** p < 0.0001, ns not significant. P values were calculated with an ordinary one-way ANOVA followed by Dunnett's multiple comparison test, with a single pooled variance.

**GATA6+ LPMs robustly take up siRNA-Cy5.5 (C12-200):** To characterize uptake of siRNA-Cy5.5 (C12-200) in LPMs, peritoneal lavage was harvested post 6 h and 24 h intraperitoneal siRNA-Cy5.5 (C12-200) administration (Figure 1A). Flow cytometry analysis revealed a significant increase in the Cy5.5 mean fluorescence intensity (MFI) as well as percentage of Cy5.5+ macrophages upon treatment of siRNA-Cy5.5 (C12-200) in the F4/80^hi^+ CD11b^hi^+ gated population (Figure 1B-D). Moreover, this was further validated by immunocytochemical analysis of the isolated LPMs which suggested a robust uptake of siRNA-Cy5.5 (C12-200) post 6 h treatment that decreased by 24 h in GATA6+ LPMs (Figure 1E, F). Although there was a reduction in fluorescence intensity by 24 h, it was still significantly higher than mice treated with PBS-controls and still demonstrated sufficient uptake of the siRNA-Cy5.5 (C12-200) into the LPMs by 24 h as also seen with MFI from flow cytometry (Figure 1C). This could possibly be due to some fluorophore quenching of the Cy5.5 fluorophore over time 41, 42.

**Figure 2:**
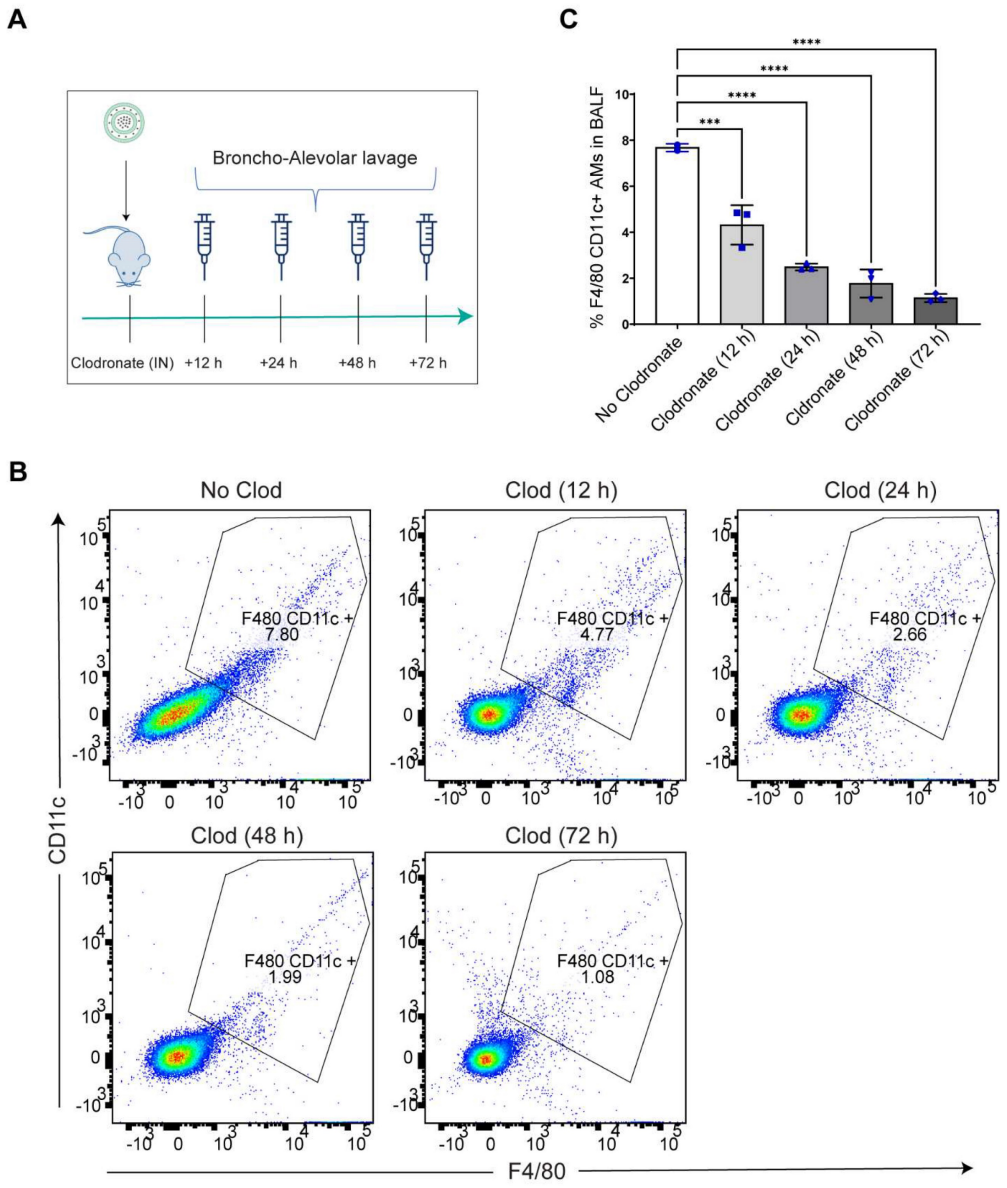
** AMs are depleted after intranasal clodronate administration. A.** Study schematics of intranasal clodronate-mediated AM depletion. **B.** Representative Flow cytometry analysis of F4/80^hi^ CD11c^hi^ large resident macrophages in the isolated BALF samples. Cells were pre-gated on size and viability. Data is representative of one sample from an n = 4 per treatment group. **C.** Quantification of flow cytometry analysis depicting a percentage of F4/80^hi^ CD11c^hi^ macrophages in the BALF samples from respective treatment groups. n = 4 for all other groups. Data has been represented as Mean +/- SEM *p < 0.05, **p < 0.01, ***p < 0.001, **** p < 0.0001, ns not significant. P values were calculated with an ordinary one-way ANOVA followed by Dunnett's multiple comparison test, with a single pooled variance.

**Intranasal administration of clodronate liposomes causes depletion of lung resident AMs:** Intranasal administration of clodronate liposomes has been well characterized to selectively deplete the lung resident AMs 27, 32. Since we wanted to explore whether selective depletion of AMs led to LPM migration to the lungs, we aimed to selectively deplete the AMs by intranasally administering clodronate liposomes at a known cytotoxic dose of 5 mg/kg 27, 32. A dose of 5 mg/kg clodronate liposomes and an equivalent concentration of blank (no clodronate) liposomes were intranasally administered to mice, and broncho-alveolar lavage fluid (BALF) was isolated at 12 h, 24 h, 48 h and 72 h post clodronate administration to assess large macrophage depletion (Figure 2A). Flow cytometry analysis revealed a time-dependent reduction in the percentage of F4/80^hi^+ CD11c^hi^+ gated population with a significant reduction in the total number of large resident macrophage population at all time points Figure S2 (Figure 2B, C). The results confirmed that there was a significant reduction in lung resident AMs.

**Figure 3:**
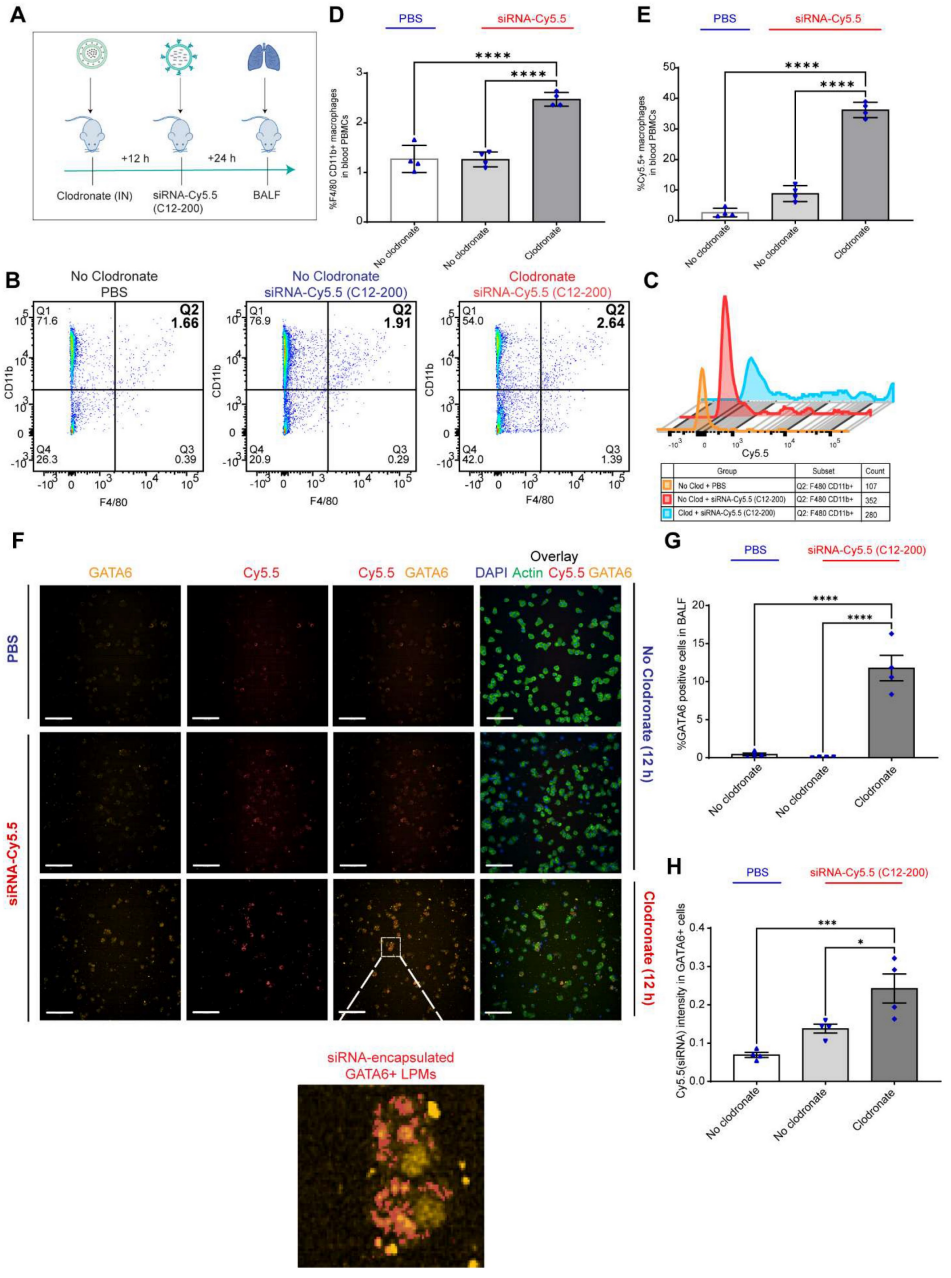
** GATA6+ siRNA-Cy5.5 (C12-200) labeled LPMs migrate to the lungs after clodronate-induced AM depletion. A.** Study schematics **B.** Representative flow cytometry dot plots of F4/80^hi^ CD11b^hi^ macrophages in BALF. **C.** Representative histograms of Cy5.5+ cells within the F4/80^hi^ CD11b^hi^ macrophage population with the indicated treatment groups. Cells were pre-gated on size and viability. Data is representative of one sample from an n = 4 per treatment group.** D.** Quantification of F4/80^hi^ CD11b^hi^ macrophage population from the flow cytometry analysis of BALF for the respective treatment groups** E.** Quantification of Cy5.5+ within the F4/80^hi^ CD11b^hi^ gated macrophage population with the indicated treatment groups. n = 4 for all the groups. **F.** Representative immunofluorescence images of siRNA-Cy5.5 (C12-200) (red) labeled GATA6+ LPMs (yellow) in the isolated BALF cells with the indicated treatment groups. n = 4 for all the groups. Scale bars, 200 µm. **G.** Quantification of GATA6+ cells from the immunocytochemistry staining and analysis of isolated BALF cells with the indicated treatment groups. **H.** Quantification of Cy5.5 intensity of isolated BALF cells in all the treatment groups. Data has been represented as Mean +/- SEM *p < 0.05, **p < 0.01, ***p < 0.001, **** p < 0.0001, ns not significant. P values were calculated with an ordinary one-way ANOVA followed by Dunnett's multiple comparison test, with a single pooled variance.

**Increase in Cy5.5 positive macrophages in the BALF after clodronate administration suggests an infiltration of a unique macrophage population to the lungs:** Next, we sought to investigate whether there was any infiltration of Cy5.5-loaded mature F4/80 expressing macrophages into the lungs at an early time point after AM depletion. We utilized flow cytometry to phenotypically characterize macrophage cell populations in the BALF. Based on prior observations, intranasal clodronate administration for 12 h already resulted in significant reduction of AM population in the lungs (Figure 2B, C). Additionally, considering the possibility of a relatively rapid migration of the LPMs to the lungs within hours after depletion of lung-resident AMs, we dosed siRNA-Cy5.5 (C12-200) intraperitoneally for 24 h following a 12 h 5 mg/kg intranasal administration of clodronate liposomes (Figure 3A). Flow cytometry analysis revealed a significant increase in the percentage of F4/80^hi^ CD11b^hi^ macrophages within the lungs only upon prior clodronate treatment of 12 h (Figure 3B, D). This was seen despite a reduction in the lung resident F4/80^hi^ CD11c^hi^ AMs as previously seen (Figure 2B, C). Upon further gating on these F4/80^hi^ CD11b^hi^ macrophages to look for Cy5.5+ cells, there was also a significant increase in Cy5.5+ macrophages seen in the BALF (Figure 3C, E). This indicated that depleting AMs might in fact be increasing infiltration of a unique Cy5.5-labeled macrophage population, especially as the siRNA-Cy5.5 (C12-200) was injected intraperitoneally for 24 h post clodronate administration. Since we previously confirmed a robust and selective uptake of siRNA-Cy5.5 (C12-200) to LPMs as early as 6 h post administration (Figure 1B-G), we next asked whether these infiltrating siRNA-Cy5.5 (C12-200)-labeled macrophages were indeed LPMs that had migrated to the lungs.

**GATA6 expressing LPMs are detected in the BALF after 12 h of clodronate administration:** To further confirm the presence of LPMs into the lungs following depletion of AM population, we carried out immunocytochemical analysis of isolated BALF and looked for Cy5.5-labeled-LPMs by overlaying immunofluorescence signal of Cy5.5 along with GATA6 expression. Since it has been well-known that GATA6 is selectively expressed by LPMs and not by AMs or rather by any other monocyte derived or TRM populations, we aimed to utilize this marker to confirm the identity of this unique TRM population 10, 11. Confocal imaging and immunocytochemical staining revealed a significant increase in the GATA6 stained cells in the BALF upon clodronate administration compared to the no-clodronate controls (Figure 3F, G). In fact, a strong nuclear staining of GATA6 was only seen in the BALF of clodronate-administered mice (Figure 3F). Along with this, there was a clear colocalization of Cy5.5+ GATA6+ cells (Figure 3F, H). Additionally, there was a further increase in Cy5.5 intensity within the GATA6+ cells upon clodronate administration demonstrating more migration of siRNA-Cy5.5 (C12-200)-loaded LPMs to the lungs after clodronate administration (Figure 3H). In combination, these data supported the hypothesis that LPMs migrated and infiltrated to the lungs from the peritoneal cavity post clodronate-administration.

**Figure 4:**
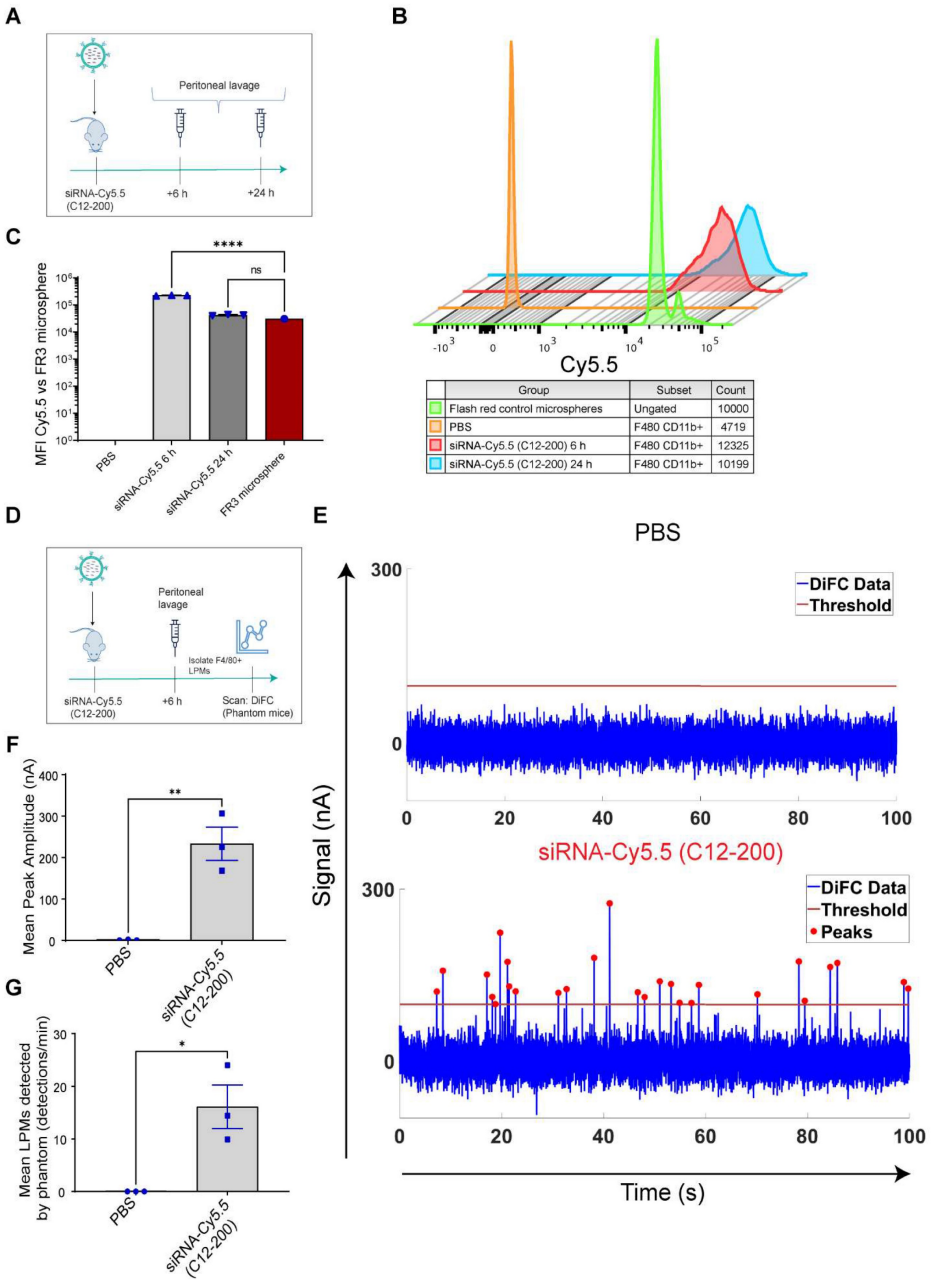
** Circulating LPMs labeled with siRNA-Cy5.5 (C12-200) are detected from peritoneal lavage by flow cytometry and DiFC in a flow phantom model. A.** Study schematics** B.** Comparative histograms of average Cy5.5 MFI from the peritoneal CD11b^hi^ F4/80^hi^ macrophages for the respective treatment groups compared to the FR3 microspheres. Cells were pre-gated on size and viability. **C.** Quantification of MFI of Cy5.5 in LPMs with the indicated groups. n = 3 for all groups except for FR3 microspheres that were run by themselves as n = 1. **D.** Study schematics for the DiFC 'phantom mouse' study post 6 h treatment with siRNA-Cy5.5 (C12-200) **E.** Representative graphs of DiFC scans depicted as number of peaks detected over time from one sample per group from an n = 3/group with the indicated treatment groups. Each peak (red circles) represents a circulating LPM labeled with siRNA-Cy5.5 (C12-200) in the peritoneal lavage F4/80^hi^ cells, depicted as signal versus time. **F.** Quantification of mean peak amplitude of all the peaks measured over time depicting the intensity of labeled circulating LPMs as detected by DiFC. **G.** Quantification of mean LPMs detected per minute as scanned by DiFC in the phantom mouse model. n = 3 for all the treatment groups. Data has been represented as Mean +/- SEM *p < 0.05, **p < 0.01, ***p < 0.001, **** p < 0.0001, ns not significant. P values were calculated with an ordinary one-way ANOVA followed by Dunnett's multiple comparison test, with a single pooled variance.

**LPMs labeled with siRNA-Cy5.5 (C12-200) *in vivo* are detected from peritoneal lavage by flow cytometry and DiFC scan in an optical flow phantom model:** We next wanted to explore the possibility of LPM intravasation into systemic circulation as one of the possible routes of how these mature TRMs migrate to the lungs upon AM depletion, and if so, track these fluorophore-labeled cells in real time to further validate our initial findings. We used a novel technique called DiFC to detect and enumerate fluorescently labeled circulating macrophages in the vasculature 36, 37. DiFC utilizes the principles of near-infrared diffuse photons to detect and count fluorescently labeled cells flowing in arteries and veins 36, 37. Hence, it makes it possible to count events as they pass through systemic circulation in a live mouse in real-time. In a series of experiments as a follow up to DiFC, we sought to confirm whether DiFC would indeed be detecting our cell-types of interest (Cy5.5-labeled circulating LPMs) with sufficient accuracy. Hence, we first tested the fluorescence intensity of siRNA-Cy5.5 (C12-200) labeled LPMs against an internal reference standard microsphere Flash Red 3 (FR3) (Bangs Laboratories Inc.) that has been previously used 37 (Figure 4A-C). We aimed to compare the MFI of LPMs loaded with siRNA-Cy5.5 (C12-200) with FR3 knowing that a higher MFI of Cy5.5 in the LPMs on the red channel would label them brightly enough to be detected by DiFC later 37. After harvesting peritoneal lavage from mice dosed intraperitoneally with siRNA-Cy5.5 (C12-200) for 6 h and 24 h, LPMs were enriched by magnetic bead depletion of non-target cells and MFI of Cy5.5 was determined using flow cytometry (Figure 4A) Figure S3A). Flow cytometry analysis revealed that the MFI of Cy5.5 of the F4/80^hi^ CD11b^hi^ gated macrophages was higher than the FR3 microspheres at both 6 h and 24 h (Figure 4B, C) (Figure S3B).

We also confirmed detection of labeled LPMs isolated from the mouse peritoneal lavage harvested after 6 h of siRNA-Cy5.5 (C12-200) treatment in a tissue simulating DiFC phantom model (37. We use this model as a proxy to confirm detection of 'peaks' of Cy5.5-loaded LPMs with accuracy as a prelude to the DiFC studies in mice *in vivo*. As previously described, the flow phantom DiFC model approximates the optical properties of biological tissue at near infrared wavelengths 36, 37. After isolated and enriched, LPMs that were previously dosed with siRNA-Cy5.5 (C12-200) were run through the phantom.

Not only was the mean peak amplitude sufficiently high in the siRNA-Cy5.5 (C12-200) treated group (Figure 4E, F) but there was also a significant increase in the number of 'peaks' that were detected (Figure 4E, G), almost surely pointing to detection of labeled LPMs in this pure sample set of harvested and enriched LPMs from mice. This confirmed that LPMs were sufficiently fluorophore-labeled with a bright enough intensity for detection in mice *in vivo* with DiFC.

**Figure 5:**
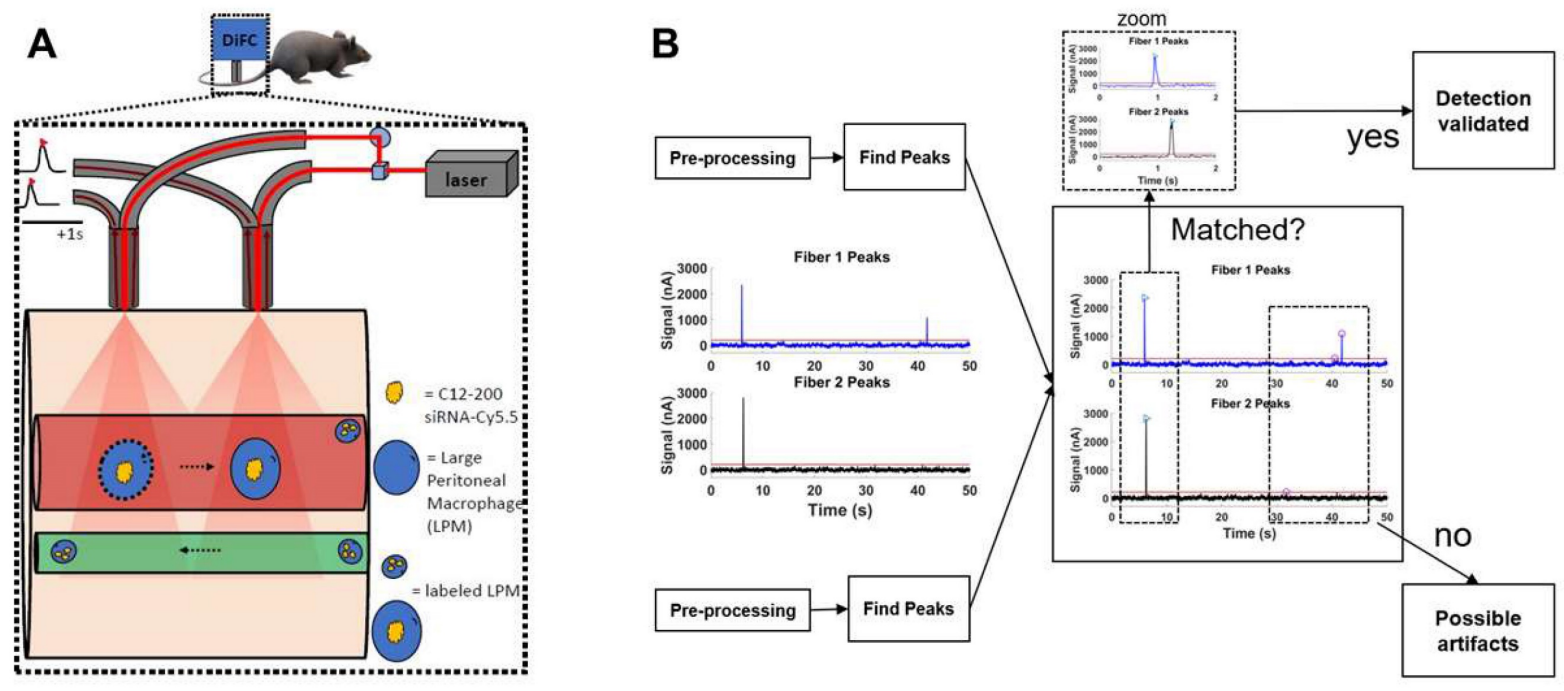
** DiFC design, schematics and analysis workflow. A.** DiFC design for mouse scanning and detection of LPM in systemic circulation. Refer text for more details.** B.** DiFC data analysis workflow.

**Figure 6:**
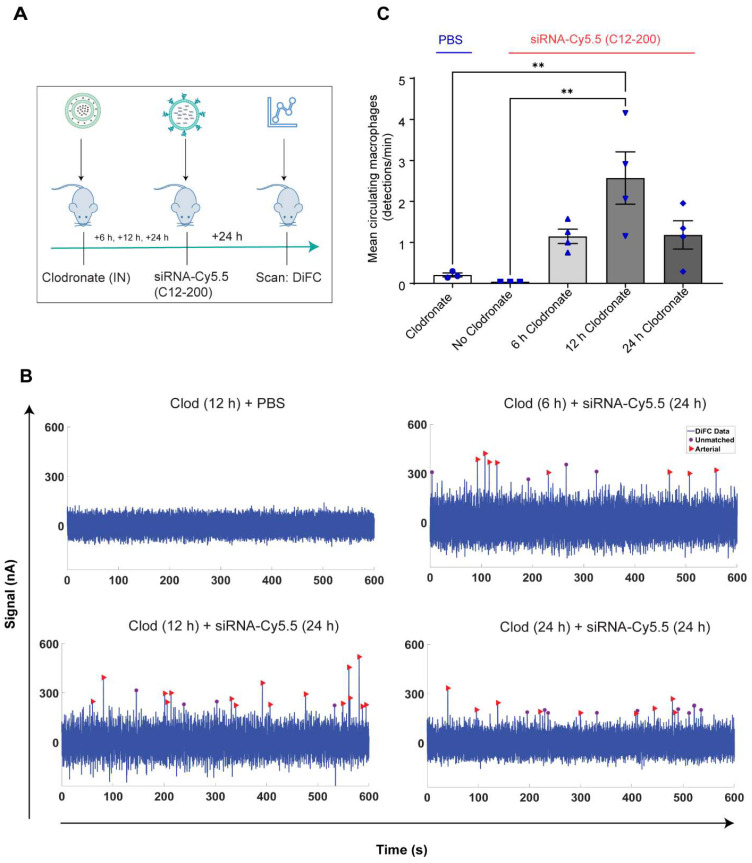
** Labeled LPMs are systemically detected by DiFC upon clodronate mediated depletion of AMs. A.** Study schematics of DiFC mouse studies post clodronate + siRNA-Cy5.5 (C12-200) administration **B.** Representative graphs of DiFC scans depicted as number of peaks detected over 600 s from one mouse per group from an n = 4/group with the indicated treatments. Graphs are representative snapshots of a 10 min scan period from a total scanning time of 45 min per mouse. Each peak (arrowhead) represents a circulating cell labeled with siRNA-Cy5.5 (C12-200) in systemic circulation, depicted as signal versus time. **C.** Quantification of mean circulating macrophages per min as detected by DiFC from a total scan time of 45 min. n = 4 for all the treatment groups. Data has been represented as Mean +/- SEM *p < 0.05, **p < 0.01, ***p < 0.001, **** p < 0.0001, ns not significant. P values were calculated with an ordinary one-way ANOVA followed by Dunnett's multiple comparison test, with a single pooled variance.

**Systemically circulating labeled PMs were detected by DiFC upon clodronate-induced AM depletion:** We next subjected siRNA-Cy5.5 (C12-200)-dosed mice to DiFC after intranasal clodronate administration. Figure 5A depicts the schematics of DiFC. As we were keen to track any labeled circulating LPMs at early time points post clodronate administration, we administered clodronate liposomes for 6 h, 12 h and 24 h to sufficiently deplete the AMs, followed by administration of siRNA-Cy5.5 (C12-200) or a dose equivalent of 1X PBS control intraperitoneally, and carried out DiFC after 24 h to give it sufficient time for the encapsulation of the siRNA-Cy5.5 (C12-200) into LPMs and intravasation of the fluorophore-labeled LPMs (Figure 6A). DiFC was performed on live mice in quadruplicate for 45 min, allowing enumeration of labeled macrophages in systemic circulation (Figure 5A, 6B) Figure S4A-D). Detection of Cy5.5 encapsulated macrophages was indicated by a transient 'peak' as cells passed through the DiFC field of view, thereby indicating the presence of LPMs in the blood (Figure 5B, 6B, C) (Figure S4A-D). Importantly, these peaks were not observed in PBS-only and no-clodronate liposome controls. The number of LPMs in circulation peaked at 12 hours following clodronate administration (Figure 6C). This suggested that LPMs intravasated following intranasal clodronate administration, and possibly migrated from the peritoneal cavity to lungs using the systemic route. It was also seen that maximum intravasation happened at 12 h post clodronate administration, further pointing out that this phenomenon of LPM intravasation and migration was acutely driven.

**Figure 7:**
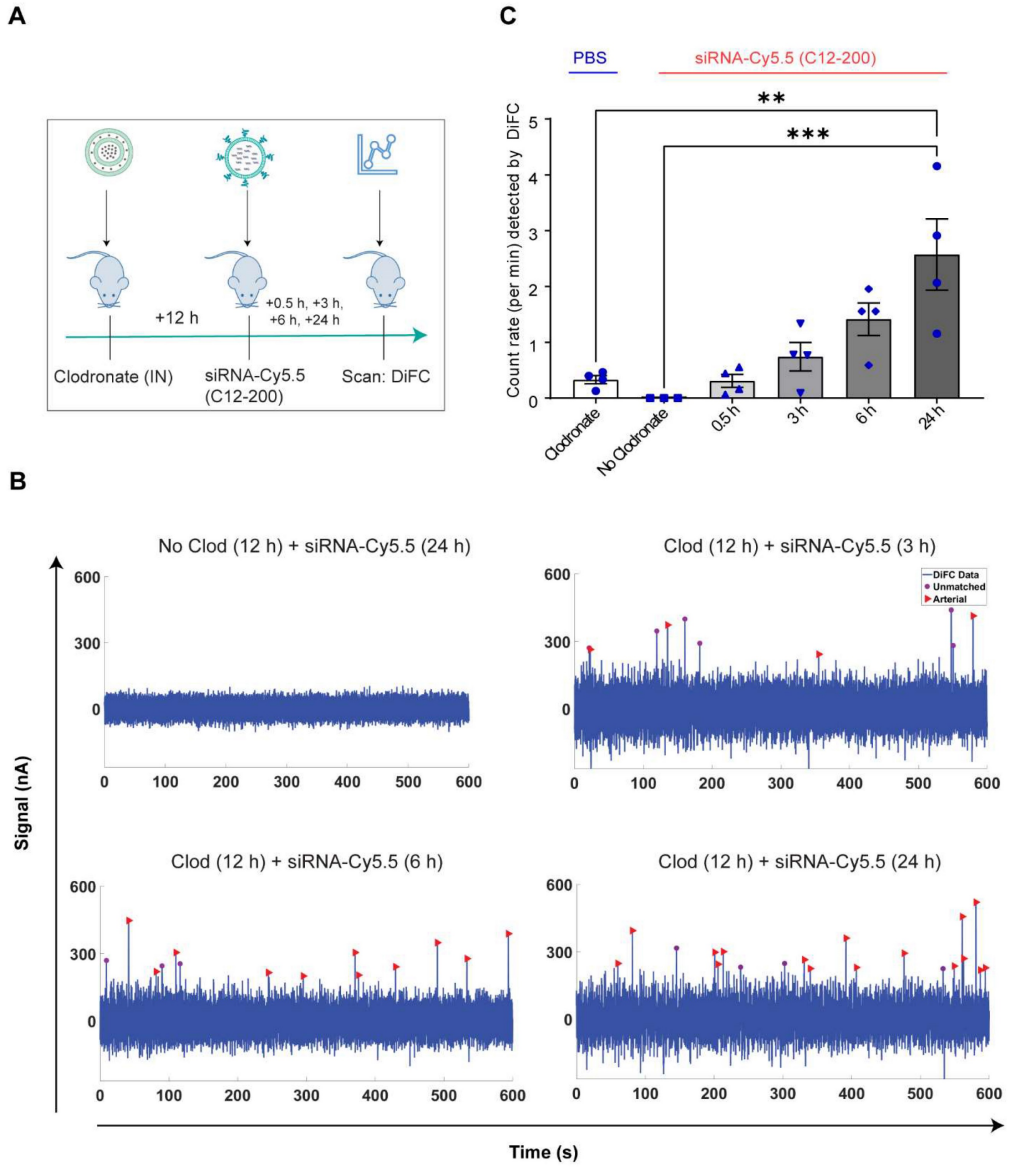
** Circulating macrophages detected by DiFC scan at different time points post 12 h clodronate administration. A.** Study schematics. **B.** Representative graphs of DiFC scans depicted as number of peaks detected over 600 s from one mouse per group from an n = 4/group with the indicated treatments. Graphs are representative snapshots of a 10 min scan period from a total scanning time of 45 min per mouse. Each peak (arrowhead) represents a circulating cell labeled with siRNA-Cy5.5 (C12-200) in systemic circulation, depicted as signal versus time. **C.** Quantification of mean circulating macrophages per min as detected by DiFC after quantifying 'matched cellular peaks' from a total scan time of 45 min. n = 4 for all the treatment groups. Data has been represented as Mean +/- SEM *p < 0.05, **p < 0.01, ***p < 0.001, **** p < 0.0001, ns not significant. P values were calculated with an ordinary one-way ANOVA followed by Dunnett's multiple comparison test, with a single pooled variance.

**Increase in number of circulating LPMs was detected by DiFC scans across different time points over 24 h post 12 h clodronate administration:** After confirming an active migration of LPMs to the lungs upon administration of clodronate liposomes, we also considered the kinetics of LPM numbers in circulation following clodronate administration. We performed DiFC at 30 min, 3 h, 6 h and 24 h after intraperitoneally injecting 1 mg/kg siRNA-Cy5.5 (C12-200) and (Figure 7A) (Figure S5A-D). In all cases, the siRNA-Cy5.5 (C12-200) injection was performed 12 h after clodronate treatment since that was when we saw the greatest number of labeled circulating 'peaks' (Figure 6C). Each DiFC scan was performed for 45 min and repeated 3 to 4 times. A progressive increase in detected circulating LPMs was observed from 30 min to 24 h, with maximum observed 24 h post siRNA-Cy5.5 (C12-200) injection (Figure 7B, C). No clodronate-liposome treated mice produced negligible detections (Figure 7C). In combination, these further validated that intravasation of labeled LPMs into systemic circulation followed clodronate-mediated AM depletion.

**Figure 8:**
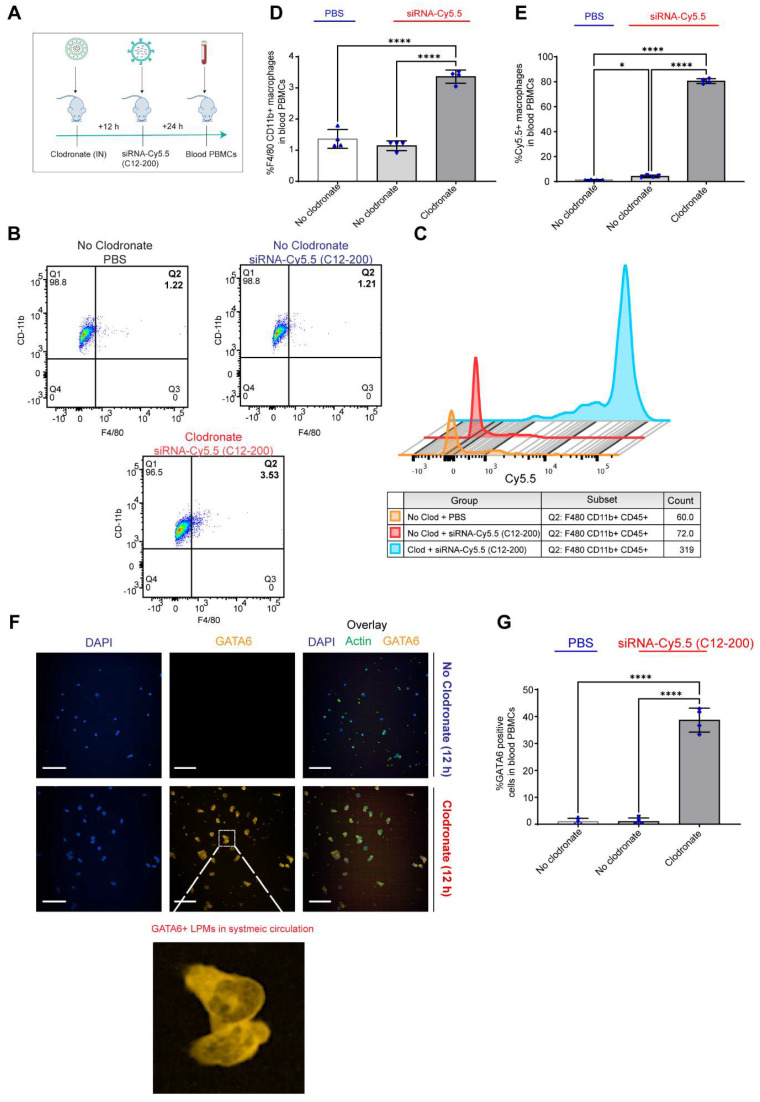
** Circulating LPMs in whole blood PBMCs are seen upon clodronate-induced AM depletion. A.** Study schematics **B.** Representative flow cytometry analysis of F4/80^hi^ CD11b^hi^ LPM population **C.** Representative histograms of Cy5.5+ cells within the F4/80^hi^ CD11b^hi^ LPM population. Cells were pre-gated on size and viability. Data is representative of one sample from an n = 4 per treatment group. **D.** Quantification of F4/80^hi^+ CD11b^hi^+ macrophage population from the flow cytometry analysis of blood PBMCs for the respective treatment groups** E.** Quantification of Cy5.5+ within the CD11b^hi^ F4/80**^hi^** gated macrophage population with the indicated treatment groups. n = 4 for all the groups. **F.** Representative immunofluorescence images of GATA6+ LPMs (yellow) in the isolated whole blood PBMC lymphocytes with the indicated treatment groups. n = 4 for all the groups. Scale bars, 200 µm **G.** Quantification of GATA6+ cells from the immunocytochemistry staining and analysis of isolated whole blood PBMC lymphocytes with the indicated treatment groups. Data has been represented as Mean +/- SEM *p < 0.05, **p < 0.01, ***p < 0.001, **** p < 0.0001, ns not significant. P values were calculated with an ordinary one-way ANOVA followed by Dunnett's multiple comparison test, with a single pooled variance.

**Clodronate-induced AM depletion leads to intravasation of LPMs into systemic circulation:** Since DiFC results pointed out to the detection of LPMs in systemic circulation only when AMs were depleted, we wanted to further corroborate our DiFC results by probing into whole blood circulating immune cells to look for mature F4/80^hi^ CD11b^hi^ GATA6 expressing LPMs in whole blood. Hence, 12 h post clodronate administration, we dosed mice with siRNA-Cy5.5 (C12-200), and isolated peripheral blood mononuclear cells (PBMCs) 24 h post injection (Figure 8A). Flow cytometry analysis revealed a significant increase in mature macrophages in clodronate-treated versus no-clodronate control samples (Figure S6 (Figure 8B, D). Although still a rare population observed in whole blood, there was a significant increase in the percentage of F4/80^hi^ CD11b^hi^ cells seen only upon clodronate administration (Figure 8D). Moreover, there was also a robust and significant increase in % of Cy5.5 cells in the F4/80^hi^ CD11b^hi^ gated population after clodronate administration (Figure 8C, E). This suggested that most of the Cy5.5-labeled mature macrophage population appeared in circulation only upon AM depletion. Satisfactorily differentiating the significant increase Cy5.5-labeled macrophages after AM depletion versus Cy5.5 labeled macrophages in no-clodronate controls was important to see due to the possibility of non-specific Cy5.5-labeled circulating cell population, owing inherently to some uptake of C12-200 LNPs by non-peritoneal macrophage cells in circulation (Figure 8E). Overall, these results further supported our DiFC findings. Finally, immunofluorescence staining of harvested PBMCs revealed a significant increase in GATA6+ LPMs in blood after clodronate administration (Figure 8F, G). Since blood monocytes, or any other cells of the immune system do not express GATA6, the presence of GATA6-expressing cells confirmed the presence of LPMs in systemic circulation which was directly dependent on clodronate mediated AM depletion.

In combination, this data supports the hypothesis that intravasation of siRNA-Cy5.5 (C12-200) labeled LPMs into systemic circulation followed clodronate-mediated AM depletion.

## Discussion

The peritoneal cavity is a fluid-filled serous cavity that is a rich source of naïve TRMs along with harboring a number of other immune cells including small PMs (monocyte-derived), B-cells and T-cells 43, 44. The true heterogeneity of all the immune cell populations residing in the peritoneal cavity has only recently been fully elucidated revealing unique transcriptomic profiles among TRMs belonging to the peritoneal cavity 6, 45, 46. One such unique identifying marker of LPMs is the zinc-finger transcription factor GATA6 11. GATA6-expressing LPMs have known tissue-specific roles, and it has been established that they are unique in their ability to migrate to areas of injury within the peritoneum, a phenomenon not seen with other TRMs 11-16. Our results suggest that this might be an inherent fundamental property of this mature innate immune cell population, as they are readily available to migrate also to extra-peritoneal tissues upon sensing depletion of a TRM population, in order to perhaps replenish them. Here we used a unique way to track intravasation of LPMs into systemic circulation and further shed light on their migratory property to the lungs simply by ablating lung-resident AMs. As discussed, since DiFC is non-invasive and does not require drawing blood, it could be performed continuously for extended periods of time (45 min in this case) while mice are under anesthesia and could be repeated at multiple timepoints to resolve the kinetics of the migration 36. DiFC is an optical technique that has been mainly used for cancer research, specifically detecting circulating tumor cells (CTCs) in mouse models of hematogenous metastasis 47-49. Here, we harnessed the application of DiFC in demonstrating that we could even detect rare-circulating immune cells like LPMs in real time in circulation in a context-dependent manner, in this case upon ablating lung-resident AMs. Moreover, we paired it with more conventional techniques like flow cytometry and immunofluorescent staining after macrophage-selective delivery of a cationic-lipid encapsulated fluorophore-labeled siRNA to explore a unique feature of a tissue-resident innate immune cell population.

Macrophages are important therapeutic targets considering their multiple vital roles in inflammatory diseases, autoimmune diseases, and cancer 50-53. Despite making significant progress with tissue-selective delivery with oligonucleotide therapies, there are still considerable roadblocks to selective delivery to immune cells 54-57. Our work has directly demonstrated the ability to deliver a fluorophore (Cy5.5)-labeled-siRNA to GATA6+ LPMs. Owing to the recent success of NP-based delivery systems for delivering oligonucleotide therapies, we successfully utilized a novel approach of encapsulating modified double-stranded siRNA in a cationic lipid, C12-200 32, 58. Future work would also explore the use of RNAi-mediated gene silencing as a therapeutic modality for macrophage delivery.

While it had been sufficiently established until now that LPMs are not necessarily 'resident' and can migrate and infiltrate peritoneally located organs like the liver and intestines via an avascular route, our results open the possibility of seeing this more broadly across non-peritoneally located organs as well and teases the question of whether this is an inherent property of these unique TRM population 13-15.

In future we plan to study the translatability of this phenomenon in multiple models of injury in multiple organs and tissues, and whether it is also translatable to higher species. Our work provides an opportunity to potentially develop RNAi therapies targeted to LPMs without the need to isolate and engineer them *ex vivo* and utilize these cells themselves as delivery modalities.

Although we do not exclude the possibility of multiple pathways and routes of migration and possibly other cell types besides LPMs that would take up the siRNA-Cy5.5 (C12-200) encapsulated liposomes, our findings do broaden the possibility of utilizing these siRNA carrying LPMs themselves as delivery vehicles especially once the specific cues or cytokines/chemokines that draw them to the lungs are identified. This could have a huge therapeutic potential, especially by combining LNP-encapsulated siRNA-mediated silencing within LPMs and modulating their migratory ability to tissues.

## Methods

**Study protocol approval:** All investigations in live mice were carried out in accordance with the Institutional Animal Care and Use Committee (IACUC) at Northeastern University and Alnylam Pharmaceuticals and conformed to the NIH guidelines for the care and use of laboratory animals.

***In vivo* studies in mice:** Balb/c mice were obtained from Charles River Laboratories. All mice were on the Balb/c background. Animals were maintained in a specific pathogen-free environment with ad libitum access to food and water. Mice were housed under standardized conditions of temperature (21-22 °C) and illumination (12/12 h light/dark cycle). Mice of 8-12 weeks of age were used for experiments. Mice were gender-matched for experiments and experimental/control mice were bred separately.

**Antibodies and reagents:** Antibodies against CD11b^hi^ and CD11c^hi^ conjugated to PE (Monoclonal Antibody M1/70, PE, eBioscience™, 12-0112-82) (Monoclonal Antibody N418, PE, eBioscience™12-0114-82) (1:100 dilution), CD45^hi^ conjugated to BUV395 (BD Biosciences, AB_2651134, 30-F11) (1:100 dilution) and F4/80^hi^ conjugated to FITC (Monoclonal Antibody BM8, FITC, eBioscience™, 11-4801-82) (1:100 dilution) (for both flow and immunocytochemistry) were obtained from eBioscience™, antibodies for Fc block (anti-CD16/CD32 Mouse BD Fc Block™; 2.4G2 clone; diluted 1-2:200, 0.5-1 ug) was obtained from BD Biosciences. Antibodies against GATA6 (D61E4 XP^®^ Rabbit mAb #5851) (1:50 dilution) and secondary antibody against the GATA6 Rabbit mAb (Anti-rabbit IgG (H+L), F(ab')_2_ Fragment (Alexa Fluor^®^ 555 Conjugate) (1:1000 dilution) #4413 were obtained from Cell Signaling Technologies. Antibodies against actin (Alexa Fluor™ 488 Phalloidin, A12379) (1:5000 dilution) were obtained from Invitrogen™. NucBlue™ Live ReadyProbes™ Reagent (Hoechst 33342) (1:10,000 dilution) was used for staining the nuclei. Clodronate liposomes as well as no clodronate control liposomes were obtained from Liposoma BV (CP-005-005).

**Synthesis of siRNA targeting CD-45 and conjugation of Cy5.5:** Double-stranded small-interfering RNA targeting CD-45 was synthesized at and provided by Alnylam Pharmaceuticals. Standard phosphor-amidite chemistry was used for siRNA synthesis. Chemical modifications were applied to the siRNA template, involving -O-methyl groups at the 2'- positions, and 2'-fluoro- groups at positions 2, 6, 14 and 16 of the antisense strand, and 7, 8, 9 and 10 of the sense strand along with capping the ends with 6 phosphorothioates (PSs) for protection from endonuclease- and exonuclease-mediated siRNA cleavage, respectively 32-34. Deprotection and purification of the crude oligoribonucleotides by anion exchange high-performance liquid chromatography were carried out according to established procedures. siRNA targeting CD-45 mRNA target site (Accession # NM_001111316.2) was generated by annealing equimolar amounts of complementary sense and antisense strands 32. A Cy5.5 fluorophore was labeled on the 5'-end of the sense strand before formulating the siRNA into NPs.

**Synthesis and characterization of C12-200-based LNPs and siRNA encapsulation:** LNPs were prepared as described previously 40. C12-200, 1,2-distearoyl-snglycero-3-phosphocholine (DSPC), cholesterol, and polyethylene glycol dimyristoyl glycerol (PEG-DMG) were dissolved in ethanol and mixed at a molar ratio of 50/10/38.5/1.5. The lipid solution was mixed with aqueous buffer containing siRNA via microfluidic mixing at a 1:3 ratio (Precision Nano systems, NanoAssemblr Benchtop Instrument). The ethanol was then removed via buffer exchange in phosphate buffered saline (PBS, pH 7.2) using dialysis. The particle size was determined using a Malvern Zetasizer NanoZS (Marlven, UK). Total siRNA content was determined by ion exchange high-performance liquid chromatography (Agilent) using DNAPac PA200 column (Dionex Corporation Dionex, 260 nm, 55 °C run at 2 ml/min). siRNA encapsulation efficiency was determined by the Quant-iT RiboGreen RNA assay (Invitrogen). Briefly, siRNA entrapment was determined by comparing the signal of the RNA-binding dye RiboGreen in formulation samples in the absence and presence of the detergent Triton-X100 (2%).

**DiFC in a flow phantom model *in vitro*:** To estimate DiFC signal detectability in mice, we first used a flow phantom *in vitro* that approximates the optical properties of biological tissue 37. The phantom is made of high-density polyethylene material and has a hole drilled 0.75 mm beneath the surface to simulate the tail artery of a mouse. We thread microbore Tygon tubing (Small Parts, Inc.) through the phantom and pump, using a syringe pump (Harvard Apparatus), a liquid suspension of fluorescently labeled LPMs at a flow rate of 50 µL/min. To determine the false alarm rate, we also used a suspension of PBS as a control.

### Experimental studies in mice

*Mouse model of clodronate-induced alveolar macrophage ablation:* For all the studies involving clodronate liposomal administration, mice were briefly anaesthetized by 2% isoflurane, and under the influence of mild anesthesia, 5 mg/kg of clodronate liposomes were intranasally administered. Likewise, 5 mg/kg of blank no clodronate liposomes that were used as controls were similarly injected. Depending on the study paradigm, clodronate/no clodronate liposomes were administered for 6 h, 12 h, 24 h, 48 h and 72 h as per the study designs.

*Administration of LNPs:* For all the studies involving administration of siRNA-Cy5.5 (C12-200), 1 mg/kg of the siRNA concentration were administered intraperitoneally for the respective treatment periods as described in the study designs.

*For DiFC studies:* For the studies involving DiFC, mice were anaesthetized with 2% isoflurane to reduce motion and kept under nosecone anesthesia to achieve a steady state of anesthesia. After shaving off the tail hair, optical fiber probes were then placed on the surface of the tail's vascular bundle along with ultrasound gel to minimize index of refraction mismatch. Heating pads were used to preserve blood circulation to the extremities. Mice were scanned for 45 min, which, based on the flow rate of the tail vasculature, allowed us to interrogate the whole peripheral blood volume of the mouse several times.

**DiFC study analysis:** DiFC is an emerging field in bio photonics that uses laser light coupled to two optical fiber-probes in series to non-invasively detect and count fluorescently labeled cells flowing in the vasculature of small animals without having to take blood draws 36. Each DiFC optical fiber probe acquires real-time data and is detected by photomultiplier tubes (PMTs).

DiFC data was analyzed as described previously 36. Briefly, first, we preprocessed the data by background subtraction. Then, we calculated the noise, which we define as the standard deviation of the data. Detections that were shown had intensity spikes at least five times greater than the calculated noise, we refer to as “peaks”. Afterwards, we did some smoothing to clean up the signal. To reduce artifacts caused by motion or instrument noise, we employ a “matching” algorithm. This consists of analyzing the peak's height and width and matching it with similar peaks appearing in the second probe (Figure 5B). When DiFC detects a peak in one optical fiber probe and then a following peak is detected in the other probe, separated by a predetermined time, we call this a “matched peak” since we can deduce that this is a cell traveling in either the arterial blood (from the heart to periphery), or the venous blood (from periphery to the heart). In this study, we observed a low percentage of matched peaks, which is likely attributed to small misalignments between both fibers on the flowing macrophage target. Because of this, we present the mean peak count rates and mean peak amplitudes of “Fiber 1” probe, which had the most peaks detected overall.

**Peritoneal lavage isolation:** Studies where peritoneal lavage was isolated, mice were sacrificed by respiratory depression under 5% isoflurane, followed by cervical dislocation following the IACUC guidelines, and peritoneal lavage was isolated following the procedure described previously 59. After peritoneal lavage isolation, LPMs were enriched following the steps from the peritoneal macrophage isolation kit (Miltenyi Biotec, Cat number 130-110-434) by magnetically labeling and depleting non-macrophage cells from peritoneal lavage and enriching for LPMs. Enriched LPMs were washed in ice-cold sterile PBS twice and resuspended in ACK lysis buffer (Gibco™ A1049201) to lyse residual red blood cells. LPMs were finally resuspended in ice cold sterile PBS. On average, after enrichment, a yield of 1×10^6^ cells were obtained in the end. These cells were then used for downstream assays.

**BALF isolation:** Studies where BALF was isolated, mice were sacrificed by respiratory depression under 5% isoflurane, followed by puncturing the diaphragm following the IACUC guidelines, and broncho alveolar lavage was isolated following the procedure described previously 60. BALF cells were washed in ice-cold sterile PBS twice and resuspended in ACK lysis buffer to lyse residual red blood cells. Finally, cells were resuspended in cold sterile PBS and counted. On average, a yield of 5×10^5^ alive cells were obtained. These cells were then used for downstream assays.

**PBMC isolation:** Studies where PBMCs were isolated, mice were put under anesthesia under 5% isoflurane, and blood was harvested by retro-orbital (RO) bleeds. This was followed by increasing the isoflurane in the chamber until induction of respiratory depression, followed by puncturing the diaphragm following the IACUC guidelines. Blood was collected in K2 EDTA tubes to avoid any clotting and 1 mL of ACK lysis buffer was added per 100 µL of whole blood and washed a couple of times. This step was repeated until the layer of RBCs was not seen. This was followed by washing with ice-cold sterile 1X PBS, making the PBMCs ready to be further prepared for flow cytometry staining and analysis.

**Flow cytometry:** Once the cells from the peritoneal lavage and BALF were harvested (as described above), a single cell suspension was generated by pressing with a plunger of a 5 mL syringe through a 70 µm nylon mesh filter into a 50 mL conical tube and washing the cells through with 5-10 mL of PBS/FCS buffer. Finally, the cells were washed in cold sterile PBS, and resuspended in FACS buffer (eBioscience™ Flow Cytometry Staining Buffer, 00-4222-57). 50 µL of cell suspension (equivalent to 10^5^ cells) was aliquoted in wells of a sterile 96-well U-bottomed plate and mixed gently by pipetting. This was followed by an addition of 50 µL of Fc block (anti-CD16/CD32 #BE0307; 2.4G2 clone; Bio X Cell, diluted 1-2:200, 0.5-1 ug) and incubated for 5-10 min to eliminate all non-specific binding. Optimal concentration was pre-determined for each antibody by priorly run pilot studies. Desired antibodies were diluted to 2 × the desired final concentration (1:200) in 100 µL of FACS buffer and added to the cell suspension, previously added to the respective wells (final Ab dilution 1:100). This was incubated for 20 min at 4^0^C in the dark, followed by washes in sterile PBS, and finally, resuspended in 100 µL of FACS buffer and ran on the BD FACSymphony^™^ A3 Flow cytometer (BD Biosciences). Later, all the data generated on the flow cytometer was analyzed using the FlowJo™ v10.8 Software (BD Life Sciences). All the flow cytometry analysis was carried out after running compensation to adjust for any fluorophore wavelength overlap, as well as pre-gating all the live events, as well as singlets before proceeding.

**Immunocytochemistry of peritoneal and broncho-alveolar lavage cells:** Once the cells from the peritoneal lavage and BALF were harvested and processed into a single cell-suspension (as described above), cells were culture in low-glucose DMEM containing 2% FBS and 1% penicillin/streptomycin at a concentration of 5×10^4^ cells per well in a 96-well tissue-culture treated plate (Cell carrier-96 ultra, 6055300) and left in an incubator (5% CO_2_, 37^0^C) overnight. Cell media was removed and 50 µL of fixative (4% PFA in PBS) (Paraformaldehyde Solution, 4% in PBS, Thermo Scientific™) was added to each well. After an incubation of 10 min at room temperature (RT), fixative was removed, and cells were washed with ice cold PBS (3×). Cells were then incubated in 50 µL per well of PTX permeabilization buffer (0.3% TritonX-100 in PBS) (diluted from Triton^™^ X-100, Sigma Aldrich, 9036-19-5) for 10 min at RT and again washed with ice cold PBS (3×) thereafter. Cells were then blocked in blocking buffer (5% NGS in PBS + 0.1% Tween-20) for an hour at RT in the dark. After aspirating the blocking solution, 50 µL per well of the primary antibody solutions were added. Primary antibody solutions were prepared by diluting Abs in PBST (PBS + 0.1% Tween-20) (information on primary Abs used described earlier under 'Antibodies and reagent' section). Cells were incubated at 4^0^C overnight in the dark, followed by washes with ice cold PBST (3×), and addition of 50 µL of secondary antibody solution (diluted in PBST similarly) and incubation for 2h at RT in the dark. After aspiration of secondary antibody, cells were washed 2× in ice cold PBST, and during the last wash, NucBlue™ Live ReadyProbes™ Reagent (Hoechst 33342) was added and incubated for 5 min. This was aspirated, and cold PBS was added followed by sealing the plate. The plate was analyzed on an Opera Phenix® High Content confocal microscope from Perkin Elmer®. Digital images were acquired using a 20X objective lens and quantification of imaged cells was carried out by the automated algorithms of the confocal microscope after optimization and background subtractions.

**Data and statistical analyses:** All the data was expressed as Mean +/- SEM. Statistical significance was determined by either unpaired student t-test (two tailed) whenever comparison between two groups was involved. In the case of 4 or more groups, one way-ANOVA (two tailed), followed by Tukey's post hoc test was used to determine statistical significance. All the experimental findings were reproduced with biological replicates of 4 unless specified otherwise, and individual experiments were reproduced twice to confirm consistency of results. A *p* value of <0.05 was considered as statistically significant. All the statistical analysis was carried out using GraphPad Prism v7.01 (GraphPad by Dotmatics, © 2022 GraphPad Software).

## Supplementary Material

Supplementary figures.

## Figures and Tables

**Table 1:**
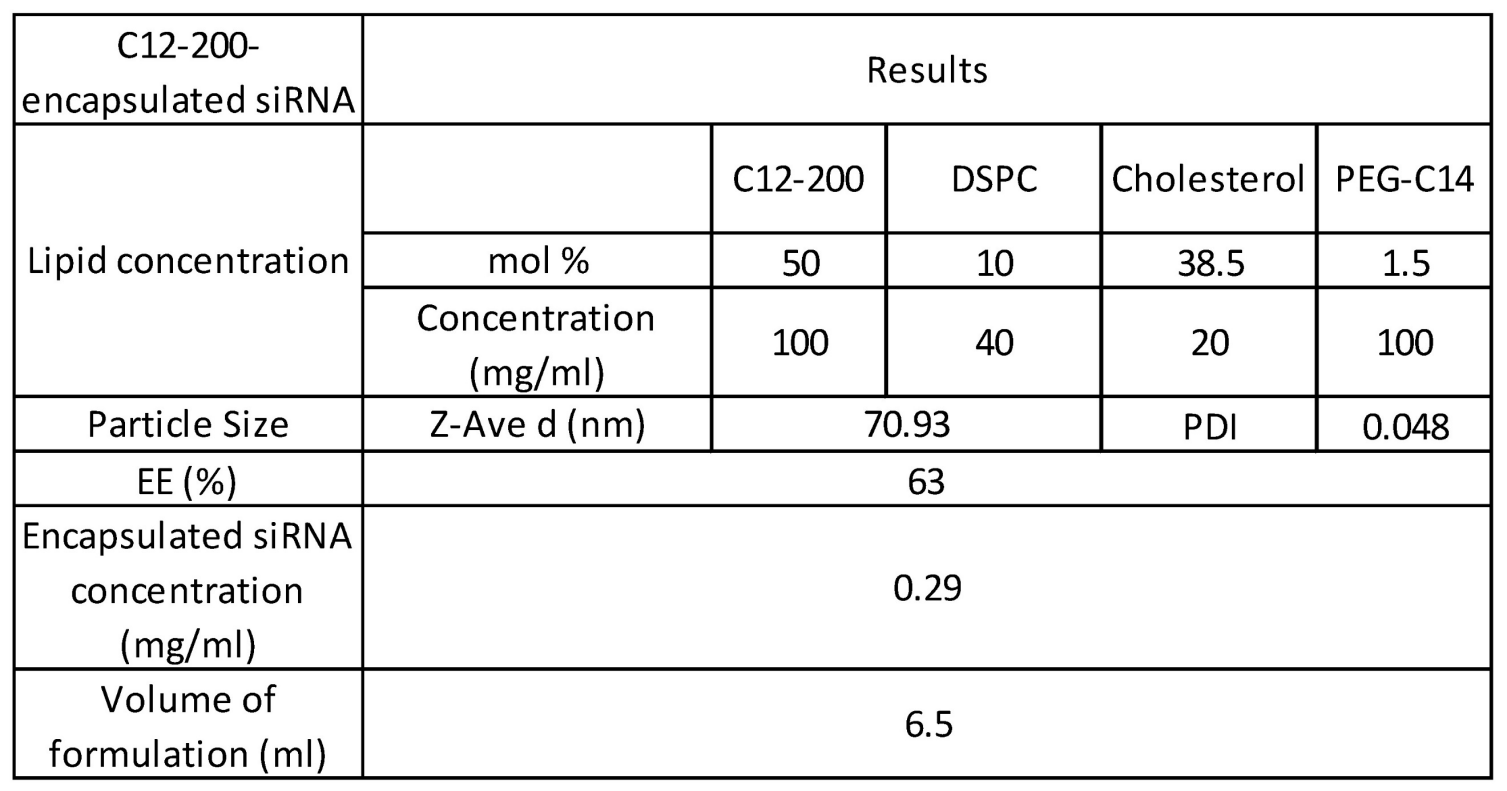
C12-200 formulation characterization, siRNA encapsulation efficiency and concentration.

Abbreviations: siRNA - small interfering RNA, DSPC - distearoylphosphatidylcholine, EE - encapsulation efficiency
