# Supplementary material for: Lipid nanoparticle encapsulated large peritoneal macrophages migrate to the lungs via the systemic circulation in a model of clodronate-mediated lung-resident macrophage depletion
Source: Theranostics. 2024 Apr 8;14(6):2526–43. doi: 10.7150/thno.91062 (PMC11024852; doi:10.7150/thno.91062)
Supplement: Supplementary file 1 — Supplementary figures. [file thnov14p2526s1.pdf]

Supplementary Information

Lipid nanoparticle encapsulated large peritoneal macrophages migrate to the lungs via the systemic circulation in a model of clodronate-mediated lung-resident macrophage depletion

Dhaval Oza[1,4], Fernando Ivich[2], Mikyung Yu[4], Mark Niedre[2], and Mansoor Amiji[1,3,5]

[1] Department of Pharmaceutical Sciences, School of Pharmacy and Pharmaceutical Sciences, 360 Huntington Avenue, Northeastern University, Boston, MA 02115

[2] Department of Bioengineering, College of Engineering, Northeastern University, 360 Huntington Avenue, Boston, MA 02115

[3] Department of Chemical Engineering, College of Engineering, Northeastern University, 360 Huntington Avenue, Boston, MA 02115

[4]Alnylam Pharmaceuticals, 675W Kendall St, Cambridge, MA, USA 02142

[5]*Corresponding Author:* Tel: 617-373-3137, email: m.amiji@northeastern.edu

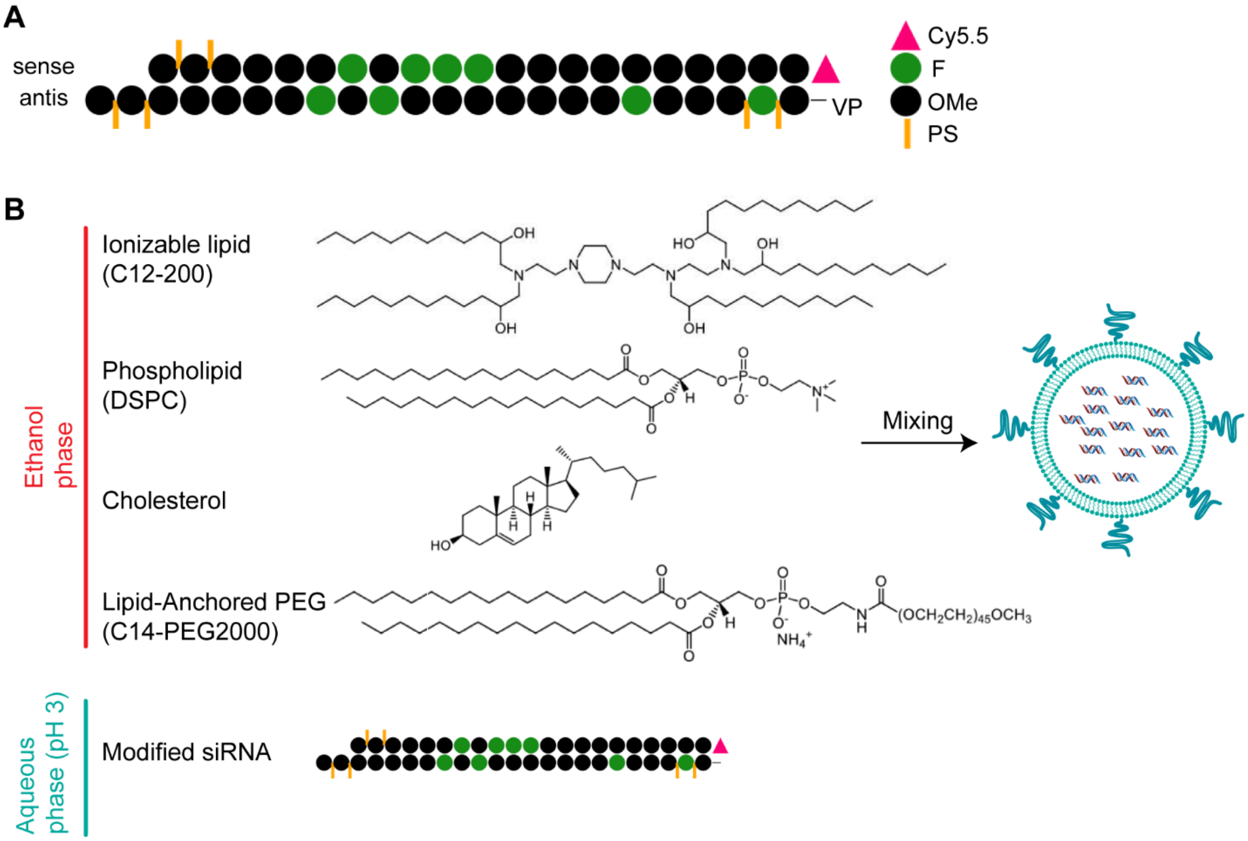

Supplementary Figure 1: siRNA design and schematics of C12-200 lipid nanoparticle formulation. A. siRNA design. A typical double-stranded siRNA design with 21 oligonucleotides on the sense strand and 23 oligonucleotides on the anti-sense strand designed against mouse CD-45 transcript, bases were modified by F- and O-Me at the 2'- positions wherever shown to avoid susceptibility to endo- and exo-nucleases. Cy5.5 was labeled to the 5'end of the sense

strand. **B.** Schematics for C12-200-based LNP formulation and siRNA encapsulation. Details described under methods and materials.

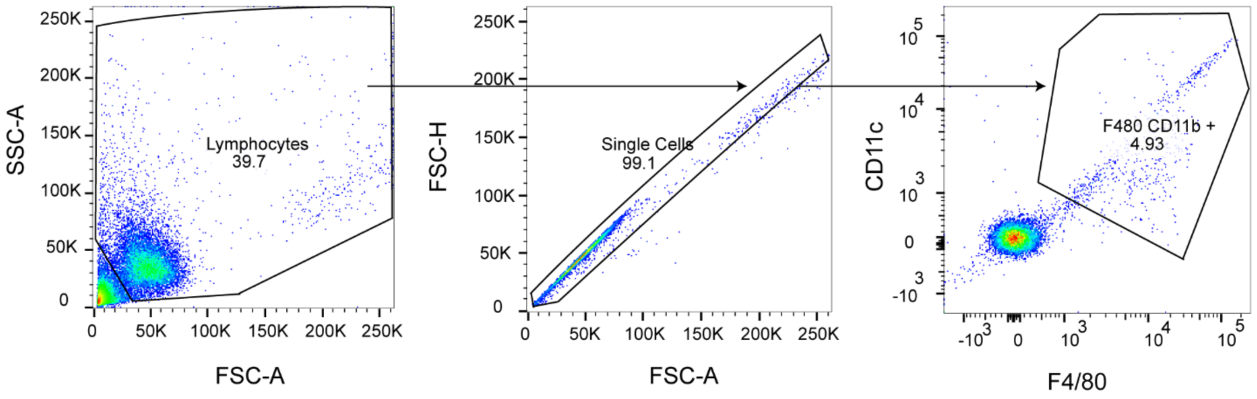

Supplementary Figure 2: Representative flow cytometry gating strategy in isolated BALF cells to assess clodronate mediated depletion of F4/80^{hi}+ CD11c^{hi}+AM population

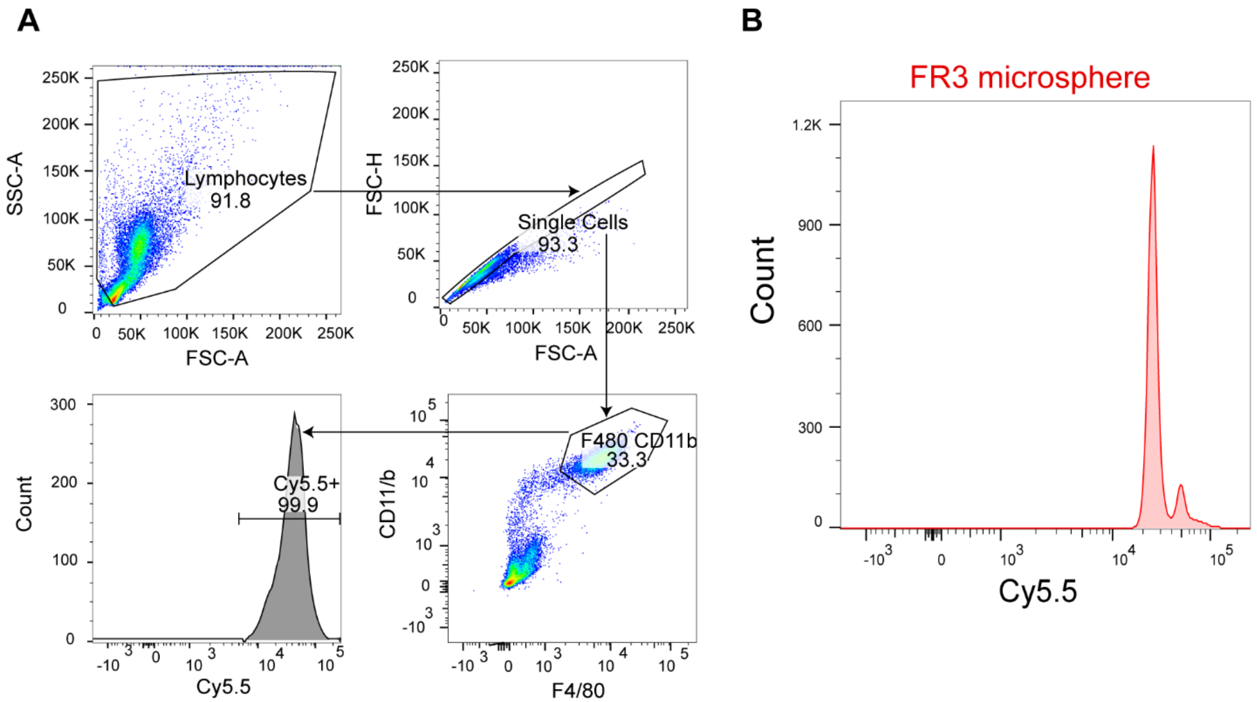

Supplementary Figure 3: Flow cytometry gating scheme of A. Cy5.5 MFI in CD11b^{hi} F4/80^{hi} LPMs obtained from the respective treatment groups along with **B.** Histogram of Flash Red 3 (FR3) microsphere MFI. Cells were pre-gated on size and viability.

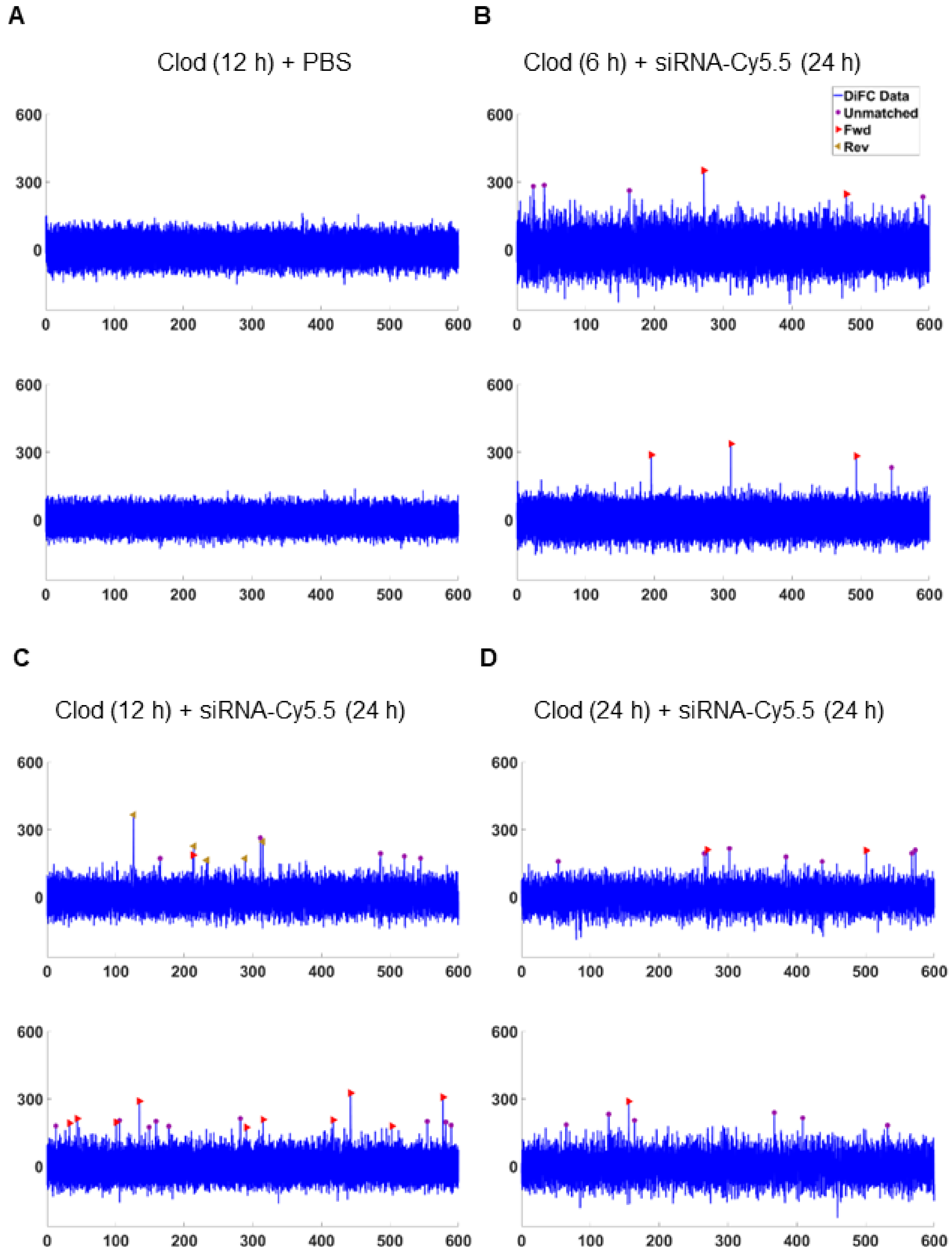

32 **Supplementary Figure 4: Representative graphs of DiFC scans for additional mice depicted**

as number of peaks detected over 600 s from 2 mice per group from an n = 4/group with the indicated treatments. A. Clod (12 h) + PBS **B.** Clod (6 h) + siRNA-Cy5.5 (24 h) **C.** Clod (12 h) + siRNA-Cy5.5 (24 h) **D.** Clod (24 h) + siRNA-Cy5.5 (24 h). The top and bottom panels are graphs for individual mice; data shown for 2 mice for the respective treatment groups. Graphs are representative snapshots of a 10 min scan period from a total scanning time of 45 min per mouse. Each peak (arrowhead) represents a circulating cell labeled with siRNA-Cy5.5 (C12-200) in systemic circulation, depicted as signal versus time.

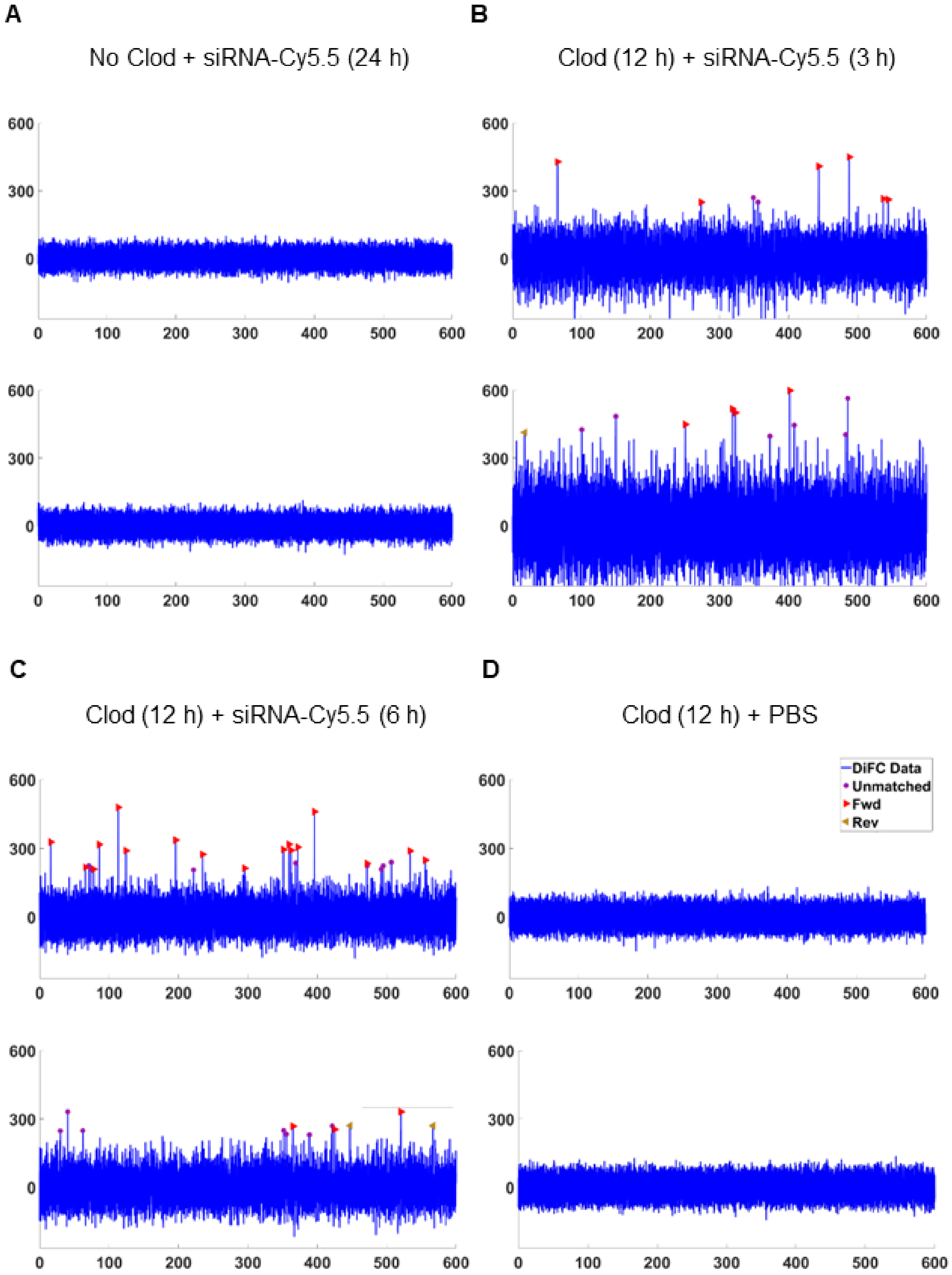
A
No Clod + siRNA-Cy5.5 (24 h)
B
Clod (12 h) + siRNA-Cy5.5 (3 h)
C
Clod (12 h) + siRNA-Cy5.5 (6 h)
D
Clod (12 h) + PBS
DiFC Data
Unmatched
Fwd
Rev

40

Supplementary Figure 5: Representative graphs of DiFC scans for additional mice depicted as number of peaks detected over 600 s from 2 mice per group from an n = 4/group with the indicated treatments. A. No Clod + siRNA-Cy5.5 (24 h) **B.** Clod (12 h) + siRNA-Cy5.5 (3 h) **C.** Clod (12 h) + siRNA-Cy5.5 (6 h) **D.** Clod (12 h) + PBS. The top and bottom panels are graphs for individual mice; data shown for 2 mice for the respective treatment groups. Graphs are representative snapshots of a 10 min scan period from a total scanning time of 45 min per mouse. Each peak (arrowhead) represents a circulating cell labeled with siRNA-Cy5.5 (C12-200) in systemic circulation, depicted as signal versus time.

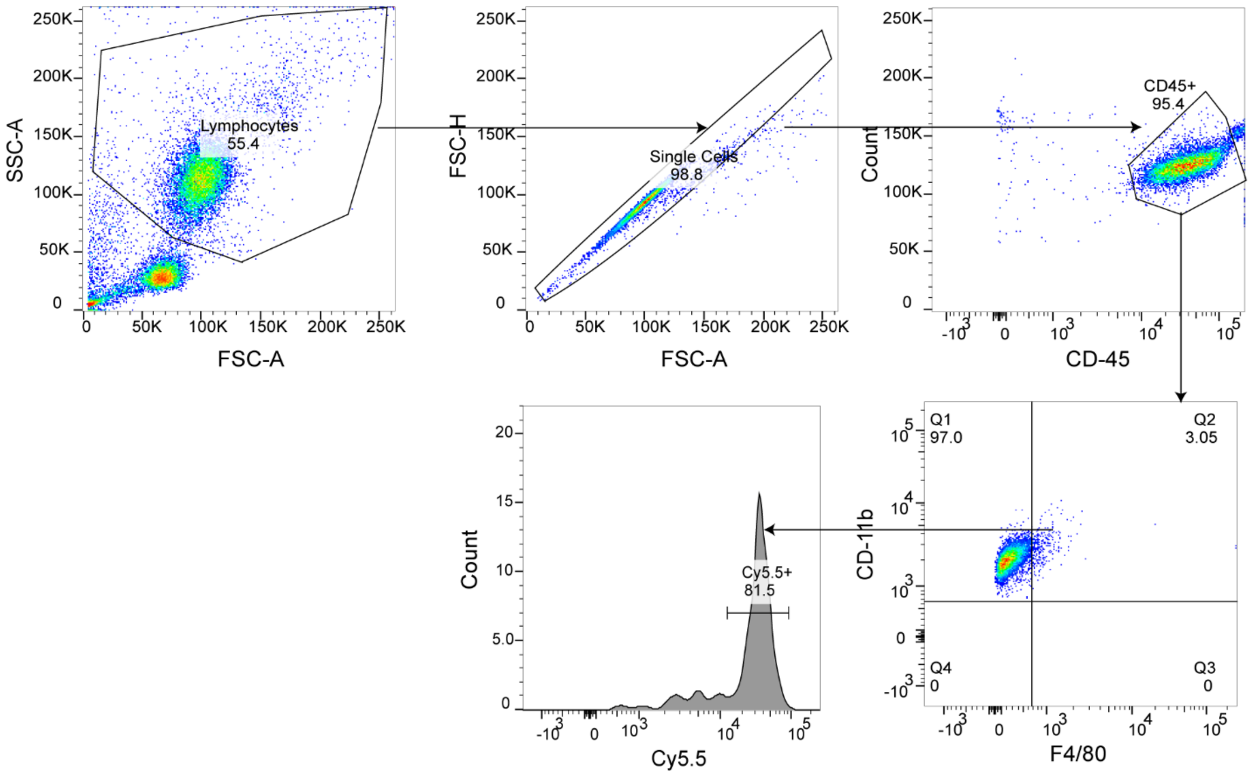

Supplementary Figure 6: Representative flow cytometry gating strategy to assess percentage of Cy5.5+ F4/80^{hi} CD11b^{hi} macrophage population in whole blood PBMCs.
